# Editorial: An integrative approach to thermoregulation

**DOI:** 10.3389/fphys.2023.1230706

**Published:** 2023-06-15

**Authors:** José Eduardo Pereira Wilken Bicudo

**Affiliations:** School of Earth, Atmospheric and Life Sciences, University of Wollongong, Wollongong, NSW, Australia

**Keywords:** temperature, ectothermic organisms, endothermic organisms, adaptive evolutionary changes, phenotypic plasticity

Temperature is one of the most relevant environmental abiotic variables, which affect living organisms in many ways. Thermoregulatory mechanisms *per se* have been the object of extensive physiological research for a long time. Now, we not only have a better understanding of how temperature is regulated at different levels of organization (from molecules, cells and organs to tissues and whole organisms) in ectothermic and endothermic organisms, but also how such organisms are affected by the outside thermal environment to maintain homeostasis. More recently, questions have been addressed in order to understand how biological traits may vary continuously as a function of temperature, and what the mechanisms underlying such traits are. Other questions that have also recently been raised are those regarding how climate variability influences plastic and evolutionary responses, and how thermoregulatory behavior alters the evolution of thermal tolerances and the impacts of climate change over short and long terms. Comparative studies at the population level reveal evolutionary differences among populations found in different geographical locations, and based on such studies we are now beginning to understand, for example, how food quality combined with temperature may also affect organisms in different ways. Unraveling the triggers, the mechanisms and the consequences of adaptive evolutionary changes in response to environment thermal variability is one of the main objectives of current biological investigation. Field-based natural history and experiments with laboratory-based biochemistry and physiology, associated with new available research tools, have allowed investigators to develop theoretical models to predict how traits (behavior, morphology and physiology) of organisms interact with climatic conditions and how these interactions affect key fitness components such as potential activity time, development and growth rates, water balance and food requirements. In addition, modern technologies combined with available databases have also been providing new knowledge of the environment in which an organism actually functions as a way to help answering those intricate questions. The aim of this special edition is not only to bring to a larger audience the current advances in the field of thermoregulation, but also to stimulate debates and further investigation in this complex research area.

Within this perspective, the review article by Favilla and Costa explores not only the thermoregulatory challenges imposed on air-breathing marine vertebrates, but also several other challenges imposed by the aquatic environment where they live. Air-breathing vertebrates must capture oxygen from the atmospheric air and deep dive to search for food. In this review we can fully appreciate the complexity of the thermoregulatory strategies involved in the temporal separation of these two conflicting situations. In response to these challenges, air-breathing vertebrates have developed morphological and physiological (including behavioral) adaptations which align with their life histories and phylogenies, thus contributing to their homeostasis.

The article by Akashi deals with the fascinating heat sensitive ion channel, the transient receptor potential ankyrin 1 (TRPA1), present in most non-mammalian species. It has been suggested that the activation of this channel induces heat avoidance behaviors in ectothermic animals. The results presented in this article support the idea that the thermal threshold of TRPA1 is well correlated with body temperatures that animals escaped from heat sources, and this finding could be used to evaluate the vulnerability of species to thermal stresses taking also into account those related to global climate warming.

Grosiak et al. investigated the relevant question whether thermoregulation in endotherms declines with age. Their working hypothesis is that the selection for an increased aerobic exercise metabolism, arguably a crucial factor involved in the evolution of endothermy in birds and mammals, may affect not only their thermoregulatory traits but also age-related changes of these traits over time. They tested this hypothesis in bank voles as a model system. Their results led them to conclude that the selection for high aerobic exercise metabolism diminished the adverse effects of aging on cold tolerance in bank voles. However, this advantageous tolerance was traded off by a compromised coping with hot conditions.

Growth, reproduction and survival are dependent on energy metabolism. To maintain a constant body temperature under continuously changing environmental conditions, such as low ambient temperatures and food availability, is challenging for endotherms. The study by Zagkle et al. was performed under the assumption that there is a positive link between aerobic metabolism and the production of oxygen reactive species. They exposed zebra finches of different age classes to food deprivation or to normal access to food at thermoneutral zone. In response to food deprivation, old birds lowered their body temperatures more than younger ones and they also showed higher oxidative damage than younger ones. Based on these results, the authors hypothesize that age-specific increase in oxidative stress is mediated by impaired thermoregulatory capacity in old zebra finches.

The thermal physiology of an amphibian host-pathogen interaction was the subject of the study carried out by Sonn et al. to make predictions of disease risk in space and time. It is well documented that the pandemic disease chytridiomycosis has been implicated in significant declines in the number of amphibious species worldwide. By using field-collected measurements of host body temperatures and other physiological parameters they were able to develop a mechanistic model of disease risk in cricket frogs, which predicts that pathogen prevalence is greatest, and survival of infected frogs is lowest, just before breeding, when host body temperatures are low. Their study clearly demonstrates that mechanistic modeling approaches are useful to predict disease outbreaks and dynamics in animal hosts.

Cold climates represented a major environmental challenge for the spread of humans out of Africa. According to Kalyakulina et al. the associated adaptive traits that allowed such spread have evolved by modulation of thermogenesis and thermoregulatory processes in which nuclear and mitochondrial genes play a major role, particularly those involved in brown adipose tissue metabolism. The study focused on three human populations (British, Finnish and Central Italian) of European ancestry from different biogeographical and climatic areas. The authors used a machine learning approach to identify relevant nuclear and mitochondrial DNA interactions characterizing each population.

